# Oral verrucous carcinoma complicating a repetitive injury by the dental prosthesis: a case report

**DOI:** 10.11604/pamj.2015.20.297.6135

**Published:** 2015-03-26

**Authors:** Laila Rahali, Youssef Omor, Karima Mouden, Youssef Mahdi, Hanan Elkacemi, Sanaa Elmajjaoui, Rachida Latib, Tayeb Kebdani, Mohamed Najib Boujida, Noureddine Benjaafar

**Affiliations:** 1 Department of Radiotherapy, National Institute of Oncology, Mohammed V Souissi University, Rabat, Morocco; 2 Department of Radiology, National Institute of Oncology, Mohammed V Souissi University, Rabat, Morocco; 3 Department of Pathology, National Institute of Oncology, Mohammed V Souissi University, Rabat, Morocco

**Keywords:** Oral verrucous, carcinoma, dental prosthesis

## Abstract

Verrucous carcinoma (VC) is an unusual, well differentiated, and low-grade type of squamous cell carcinoma, characterized by benign histology and cytology but markedly invasive clinical behavior. They have a predilection for squamous mucosae, particularly those of the head and neck region. Many factors have been associated with its pathogenesis, including the presence of previous skin lesions; VC arising from a prosthesis injury is rare. Here we reported a case of VC of oral cavity a particularly very aggressive, arising from prosthesis injury. Regardless of the treatment modality, given new insights into the possible aggressivity of this tumor, radiotherapy associated to chemotherapy may be a more appropriate primary treatment compared with the significant local morbidity associated with surgery.

## Introduction

The Verrucous Carcinoma was described by LV Ackerman in 1948 as an infrequent subtype of malignant disease of the oral cavity [1]. It is a rare tumor representing only 3-4% of all oral carcinomas [2]. The etiology and pathogenesis of OVC are not clear, though there is a strong association with the use of tobacco, alcohol, and poor oral hygiene [3]. Surgery with wide margins is considered the primary mode of treatment. We report a case particularly aggressive complicating a repetitive injury by the dental prosthesis. The objective of this work was to discuss the mechanism, diagnosis and treatment compared with literature data.

## Patient and observation

**Figure 1:**
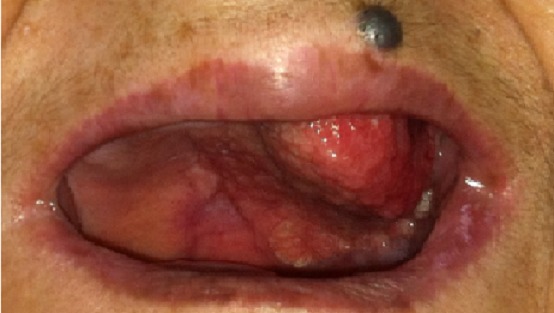
Clinical photography of oral verrucous carcinoma

**Figure 2:**
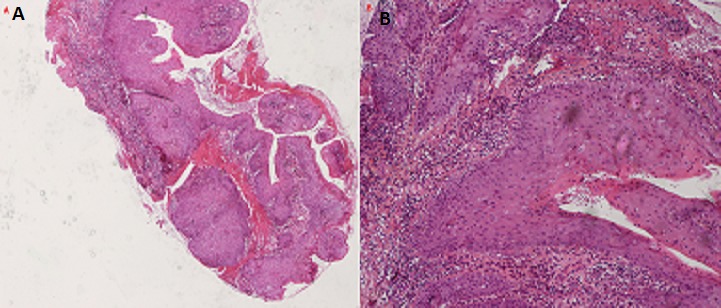
Histological aspect of the lesion: A) exophytic and endophytic marked acanthosis and hyperkeratosis (H&E, low magnification); B) minimal cytological atypia and pushing margins without a frank infiltration (H&E, high magnification)

**Figure 3:**
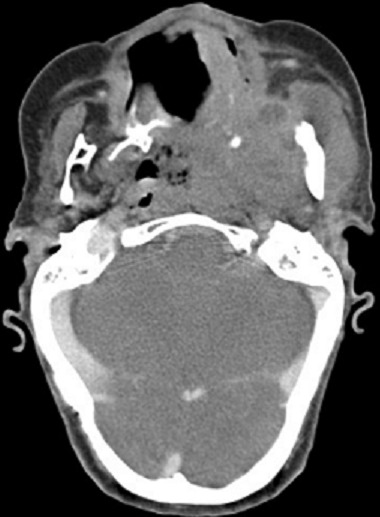
Axial post-contrast CT demonstrates tissue thickening of the hard palate extended to the masseter muscle and the nasopharynx

A 63-year-old woman was received on January 16, 2014 in INO Radiotherapy Department for oral cavity tumor. The patient had a history of repetitive injury by the dental prosthesis. Past medical history was otherwise noncontributory. She was presented with a 4-month history of a painless growing tumor of the hard palate, associated to left-sided otalgia and subjective left-sided hearing loss. She didn't have any vertigo, headaches, or vision changes. On examination a cauliflower-shaped exophytic lesion was present on the left lower alveolar ridge and gingiva extending to hard palate measuring 60×45cm (Figure 1). On palpation, the lesion was tender and margins were indurated. Teeth in that dentoalveolar segment were not mobile. Left cervical lymph nodes were enlarged. Her oropharyngeal examination was unremarkable. The biopsy was histopathologically assessed as verrucous carcinoma. The pathologists confirmed the clinic diagnosis of non infiltrative VC, on the base of these features: dense superficial keratinisation, dyskeratosis, minimal cytological atypia, pushing margins without a frank infiltration, nor vascular or perineural invasion (Figure 2). A second biopsy exhibited similar features. The head and neck CT showed (Figure 3) tissue thickening of the hard palate extended to the floor of the nasal cavity, with lysis of the maxillary sinus and extension to the masseter muscle, the nasopharynx and infratemporal fossa. The tumor is associated with submandibular nodes and upper internal jugular nodes. TNM classification was T4N1M0. The patient had received three cures of neo-adjuvant chemotherapy of 5-Fluouracil and cisplatin. The control head and neck CT showed disappearance of nodes enlargement but with the same extension of the tumor. She received external radiotherapy of 50 Gy in 25 fractions. She made an uneventful recovery. There was neither local recurrence nor distant metastasis observed for six months.

## Discussion

Verrucous carcinoma (VC) is a non-metastasing variant of well-differentiated squamous cell carcinoma (SCC), which often presents as an exophytic, warty tumor. The oral cavity is the most common site. The buccal mucosa, tongue, alveolar ridge, and lips are the sites usually involved. Oral verrucous carcinoma (OVC) typically occurs in elderly patients [2]. Previous lesions as leucoplakias or eritroplakias, as well as proliferative verrucous leucoplakia, are the sites where the OVC uses to arise from [1]. Its etiology is not well known, but smoking habit, alcohol consumption and betel nut chewing are proved causes [1]. More recent theories concerning the pathogenesis of verrucous carcinoma include nullification of a tumor suppressor gene by types of human papillomavirus [4]. Although, the role of the HPV in OVC oncogenesis is much less important than in the SCC oncogenesis [1]. Repetitive mechanical trauma may contribute to verrucous carcinoma [4]. This mechanism may parallel the trauma of poorly fitting dentures for oral lesions as found in our case. Verrucous carcinoma a warty variant of squamous cell carcinoma is characterized by a predominantly exophytic overgrowth of well-differentiated keratinizing epithelium [5]. The classical histopathological features are an intact basement membrane, with preservation of stratification and broad rete pegs of carcinoma cells, which appear to punch into the underlying tissue. The most important pathological difference with squamous cell carcinoma is the good cytological differentiation throughout the tumor. Verrucous carcinoma can also be mistaken as a benign lesion histologically. There is also a problem of so called “hybrid” tumor involving both verrucous carcinoma and the usual squamous cell carcinoma. Also the histopathological diagnosis of verrucous carcinoma is sometimes deceptive because in some cases, superficial biopsies will show only hyperkeratosis, acanthosis, and benign papillomatosis. Deeper tissue biopsies are required for proper diagnosis [6]. The lesser aggressive nature of VC establishes it as a low grade, well-differentiated variant of oral SCC with an excellent prognosis and indolent clinical behavior. Having said that, the clinical behavior of VC can be, at times, destructive despite its deceptively benign microscopic appearance [5]. Optimal treatment of OVC remains controversial, and there is no worldwide consensus Because metastases are uncommon, local control and the preservation of function and cosmesis are the main goals of treatment. Resection is the treatment of choice [2]. Histological margins above 5 mm are considered sufficient to not increase the risk of local recurrence. In the impossibility to increase resection margins in a second surgery, postoperative radiotherapy could be considered [1].

The need for a cervical dissection is also controversial. Case series published over the years agree that the OVC tends to grow locally but not to cause ganglionar spread [1]. Excision of the tumor is often possible for small lesions without compromising function or cosmesis. The character of this type of tumor is indolent, so patients often present with advanced disease. In such instances resection may not be feasible and may lead to poor functional and cosmetic results [2]. The high rate of recurrence after surgery calls into question the operative management of the disease [6]. CT of head and neck may help make the decision of the treatment choice. If there is wide spread with clear involvement of bone, muscular and vascular structures likely to result in significant postoperative morbidity if damaged during extirpation (e.g., encasement of cranial nerves) radiographic information might encourage an alternative approach. The main alternative to surgery would have been primary radiotherapy [4]. Radiotherapy is an acceptable alternative in cases where resection is not an option. However, OVC is thought to be less radiosensitive than conventional SCC, so local control after radiotherapy is therefore not as good as after operation. Subjective tolerance and sometimes compliance in older patients are significantly lower, which leads to withdrawal of the treatment even though acute and chronic side-effects of radiotherapy are comparable with those in younger patients. Anaplastic transformation of OVC has been reported after radiotherapy. However, this has also been reported after resection and in an untreated OVC and may be the result of an incorrect histopathological diagnosis [2]. The effectiveness of pre-operative chemotherapy for advanced verrucous carcinoma of the tongue has been reported. However, the effect of chemotherapy on verrucous carcinoma has not been thoroughly estimated at this moment in time [6]. Although it is associated with an excellent prognosis, VC has an intrinsic potential for local recurrence that should be considered when planning surgery. Observations show their tendency to recur in the form of less-differentiated carcinomas. Metastatic spread occurs late, if at all [5]. **Consent:** Written informed consent was obtained for publication of this case report and accompanying images.

## Conclusion

Verrucous carcinoma is a rare oral tumor. Although its histology is seemingly benign, it follows an insidious clinical course with aggressive local invasion and lymph node metastasis. The role and the effect of radiation therapy on VC is controversery. As the literature is confusing, we retain that radiotherapy could be used only in selected clinical settings, when surgery is not possible.
